# The prognostic roles of the prognostic nutritional index in patients with intraductal papillary mucinous neoplasm

**DOI:** 10.1038/s41598-020-79583-6

**Published:** 2021-01-12

**Authors:** Yukiyasu Okamura, Teiichi Sugiura, Takaaki Ito, Yusuke Yamamoto, Ryo Ashida, Katsuhisa Ohgi, Keiko Sasaki, Hiroto Narimatsu, Katsuhiko Uesaka

**Affiliations:** 1 Division of Hepato-Biliary-Pancreatic Surgery, Shizuoka Cancer Center Hospital, 1007, Shimo-Nagakubo, Sunto-Nagaizumi, Shizuoka, 411-8777 Japan; 2 Division of Diagnostic Pathology, Shizuoka Cancer Center Hospital, Shizuoka, Japan; 3 Cancer Prevention and Control Division, Kanagawa Cancer Center, Yokohama, Japan

**Keywords:** Gastroenterology, Medical research, Oncology, Risk factors

## Abstract

The preoperative accurate diagnosis is difficult in the patients with intraductal papillary mucinous neoplasm (IPMN). The aim of the present study was to elucidate the roles of systemic inflammation responses and nutritional status indexes in IPMN. High-grade dysplasia was classified as a malignant neoplasm in the study. We retrospectively reviewed 155 patients who underwent pancreatectomy. The correlation between the clinical factors and several indexes of a systemic inflammation response and nutritional status was analyzed. Among the biomarkers, prognostic nutritional index (PNI) value of malignant IPMN patients was significantly lower than that of benign IPMN patients (*P* = 0.023), whereas PNI was not significant predictor for malignant IPMN. The multivariate analysis showed that a PNI < 43.5 (odds ratio [OR] 16.1, 95% CI 1.88–138.5, *P* = 0.011) and a carbohydrate antigen (CA) 19–9 level > 22.5 U/mL (OR 6.64, 95% CI 1.73–25.6, *P* = 0.006) were significant independent predictors of the presence of lymph node metastasis (LNM). Our scoring system developed based on these two factors. Patients with a score of 0 had no LNM and zero disease-related death. The present study suggested the roles of PNI on the IPMN patients who undergo curative pancreatectomy.

## Introduction

The World Health Organization (WHO) in 2000 defined intraductal papillary mucinous neoplasms (IPMNs) of the pancreas as intraductal mucin-producing neoplasms which have characteristics with cystic dilation of the main and/or side branches of the pancreatic duct^1^ and are known to be potentially malignant cystic lesions^2^. The guidelines are useful for deciding on a treatment strategy for IPMNs because they include the role of endoscopic ultrasonography, surgical indications, and the frequency and period of follow-up surveillance imaging examinations^2,3^. Since the publication of the Fukuoka criteria^3^, several studies have evaluated the utility of these criteria^4–6^.

Invasive intraductal papillary mucinous carcinoma (IPMC) develops in IPMNs over many years in an adenoma–carcinoma sequence^7,8^. Although IPMC is staged like conventional pancreatic ductal adenocarcinoma (PDAC)^9^, the prognosis of IPMC is generally believed to be more favorable than that of conventional PDAC^10,11^. The prognostic factors of IPMC may therefore differ from those of PDAC; however, detailed reports on this issue have not been published.

In the field of conventional PDAC, a number of prognostic factors, such as carbohydrate antigen (CA) 19–9, have been reported^12^. Recently, the systemic inflammatory response and nutritional status have been recognized as closely linked to various stages of hepato-biliary-pancreatic cancer^13–18^. Biomarkers of systemic inflammation such as the neutrophil-to-lymphocyte ratio (NLR)^13,14^ and the platelet-to-lymphocyte ratio (PLR)^14^, and the nutritional status, such as the Prognostic Nutritional Index (PNI)^15^, have been associated with a poor survival of PDAC. Although the NLR and PLR have been reported to be the predictive markers of IPMC among these indexes^16–18^, no reports have described the utility of the PNI. Furthermore, most papers related to IPMNs have reported on the discrimination between benign and malignant disease^4–8,16–18^, and there are relatively few reports on prognostic factors^19–21^.

The aims of the present study were to elucidate the roles of the indexes of a systemic inflammation response and nutritional status and to identify the prognostic factors of IPMN.

## Results

### Patient characteristics

A total of 164 consecutive patients were included in the study period. Of these, 9 IPMN patients with concurrent conventional PDAC were excluded in the present study. The patients’ clinical characteristics are shown in Table 1. There were 66 patients with invasive IPMC, 31 patients with high-grade dysplasia (HGD) (carcinoma in situ) and 58 patients with benign neoplasms. No patients with a history of inflammatory disease or active concomitant infection were included.

**Table 1:** Demographics of IPMN patients.

Characteristics	Value
Age (years)^#^	68 (32–86)
Sex (males/females)	105/50
CEA (U/mL)^#^	2.3 (0.5–24.5)
CA19-9 (U/mL)^#^	10 (2.0–2,661)
Cyst diameter (mm)^#^	32 (7–140)
Main pancreatic duct diameter (mm)^#^	7.0 (1.0–50)
NLR	1.88 (0.69–13.1)
PLR	77.2 (24.9–268.0)
PNI	43.2 (22.1–52.1)
Operative indication according to the Fukuoka criteria, presence	130 (83.9)
**High-risk stigmata according to the Fukuoka criteria, presence**	106 (68.4)
Jaundice, presence	14 (9.0)
Enhanced solid component, presence	77 (49.7)
Main pancreatic duct, dilatation ≥ 10 mm, presence	52 (33.5)
**IPMN type according to the Fukuoka criteria**	
Main duct	48 (31.0)
Branch duct	68 (43.9)
Mixed	39 (25.1)
Operative procedure (PD/DP/TP/MP)	94/43/11/7
**Pathological results**	
Mild or moderate-grade dysplasia	58 (37.4)
High-grade dysplasia (carcinoma in situ)	31 (20.0)
Invasive IPMC	66 (42.6)
T factor (1/2/3)	10/14/42
N factor (0/1)	46/20
Adjuvant chemotherapy, introduced	17 (11.0)

Values in parentheses are percentages unless otherwise indicated.

Values are shown as the ^#^median (range).

*IPMN* intraductal papillary mucinous neoplasm, *CEA* carcinoembryonic antigen, *CA* carbohydrate antigen, *NLR* neutrophil-to-lymphocyte ratio, *PLR* platelet-to-lymphocyte ratio, *PNI* prognostic nutritional index, *PD* pancreaticoduodenectomy, *DP* distal pancreatectomy, *TP* total pancreatectomy, *MP* middle pancreatectomy, *IPMC* intraductal papillary mucinous carcinoma.

### The comparisons of preoperative characteristics between benign and malignant IPMN

Table 2 shows the comparisons of preoperative factors between the patients with benign and malignant IPMN. The presence rates of operative indication and high-risk stigmata according to the Fukuoka criteria were significantly higher in malignant IPMN than those in benign IPMN (operative indication, 93/97 patients [95.9%] vs. 37/58 patients [63.8%], *P* < 0.001; high-risk stigmata, 84/ 97 patients [86.6%] vs. 22/58 patients [37.9%], *P* < 0.001, respectively). Among the biomarkers of systemic inflammation and the nutritional status, PNI value of the patients with malignant IPMN (median: 42.2, range: 22.1–52.1) was significantly lower than that of the patients with benign IPMN (median: 43.7, range: 30.2–52.1) (*P* = 0.023). We also performed a comparison between patients with HGD and those with invasive IPMN among patients with malignant IPMN. The median PNI value of the patients with HGD was 44.1 (range 32.2–52.1), which was marginally higher than that of patients with invasive IPMN (median PNI: 42.2, range: 22.1–50.1) (*P* = 0.081).

**Table 2:** Comparisons of preoperative characteristics of benign and malignant IPMN patients.

	Benign IPMN	Malignant IPMN	*P*
	n = 58	n = 97	
Age (years)^#^	68 (42–78)	69 (32–86)	0.165
Sex (males/females)	37/21	93/13	0.772
CEA (U/mL)^#^	1.9 (0.5–24.5)	2.6 (0.5–17.0)	0.139
CA19-9 (U/mL)^#^	7 (2–185)	13 (2–2,661)	0.001
Cyst diameter (mm)^#^	30 (7–60)	36 (7–140)	0.015
Main pancreatic duct diameter (mm)^#^	6.0 (1.0–43.0)	8.0 (2.0–50.0)	0.067
NLR	1.74 (0.69–13.1)	1.93 (0.90–8.85)	0.070
PLR	81.5 (36.5–268.0)	72.6 (24.9–187.3)	0.191
PNI	43.7 (30.2–52.1)	42.2 (22.1–52.1)	0.023
Operative indication according to the Fukuoka criteria, presence	37 (63.8)	93 (95.9)	< 0.001
**High-risk stigmata according to the Fukuoka criteria, presence**	22 (37.9)	84 (86.6)	< 0.001
Jaundice, presence	0 (0)	14 (14.4)	0.001
Enhanced solid component, presence	7 (12.0)	71 (72.4)	< 0.001
Main pancreatic duct, dilatation ≥ 10 mm, presence	17 (29.3)	35 (36.1)	0.380
**IPMN type according to the Fukuoka criteria**			0.493
Main duct	16 (27.6)	32 (33.0)	
Branch duct	29 (50.0)	39 (40.2)	
Mixed	13 (22.4)	26 (26.8)	

Values in parentheses are percentages unless otherwise indicated.

Values are shown as the ^#^median (range).

*IPMN* intraductal papillary mucinous neoplasm, *CEA* carcinoembryonic antigen, *CA* carbohydrate antigen, *NLR* neutrophil-to-lymphocyte ratio, *PLR* platelet-to-lymphocyte ratio, *PNI* prognostic nutritional index.

Table 3 summarizes the odds ratios (ORs) for the factors that were identified in the univariate logistic regression analyses as possible predictors of malignant IPMN. In the multivariate analysis, the following factors remained significant independent preoperative predictors of malignant IPMN: the presence of high-risk stigmata according to the Fukuoka criteria (odds ratio [OR] 8.65, 95% confidence interval [CI] 3.76–19.9, *P* < 0.001), cyst diameter > 36 mm (OR 2.97, 95% CI 1.29–6.81, *P* = 0.010) and CA19-9 > 22.5 U/mL (OR 2.76, 95% CI 1.09–7.02, *P* = 0.033).

**Table 3:** Predictors of malignant IPMN among IPMN patients who underwent pancreatectomy.

Variables	Univariate analysis		Multivariate analysis	
	Odds ratio (95% confidence interval)	*P*	Odds ratio (95% confidence interval)	*P*
CA19-9 (> 22.5 U/mL)	3.99 (1.76–9.02)	0.001	2.76 (1.09–7.02)	0.033
Cyst diameter (> 36 mm)	2.86 (1.41–5.78)	0.003	2.97 (1.29–6.81)	0.010
PNI (< 42)	2.17 (1.03–4.56)	0.040		
High-risk stigmata according to the Fukuoka criteria (presence)	10.6 (4.80–23.3)	< 0.001	8.65 (3.76–19.9)	< 0.001

*IPMN* intraductal papillary mucinous neoplasm, *CA* carbohydrate antigen, *PNI* prognostic nutritional index.

### The preoperative predictors for the DSS in invasive IPMC patients

The median follow-up period after surgery was 50.5 months (range 1.1–184.9 months). There were no disease specific death in the patients with benign IPMN and HGD (carcinoma in situ) and then only invasive IPMC patients were analyzed to identify the predictors for the disease specific survival (DSS).

**Figure 1:**
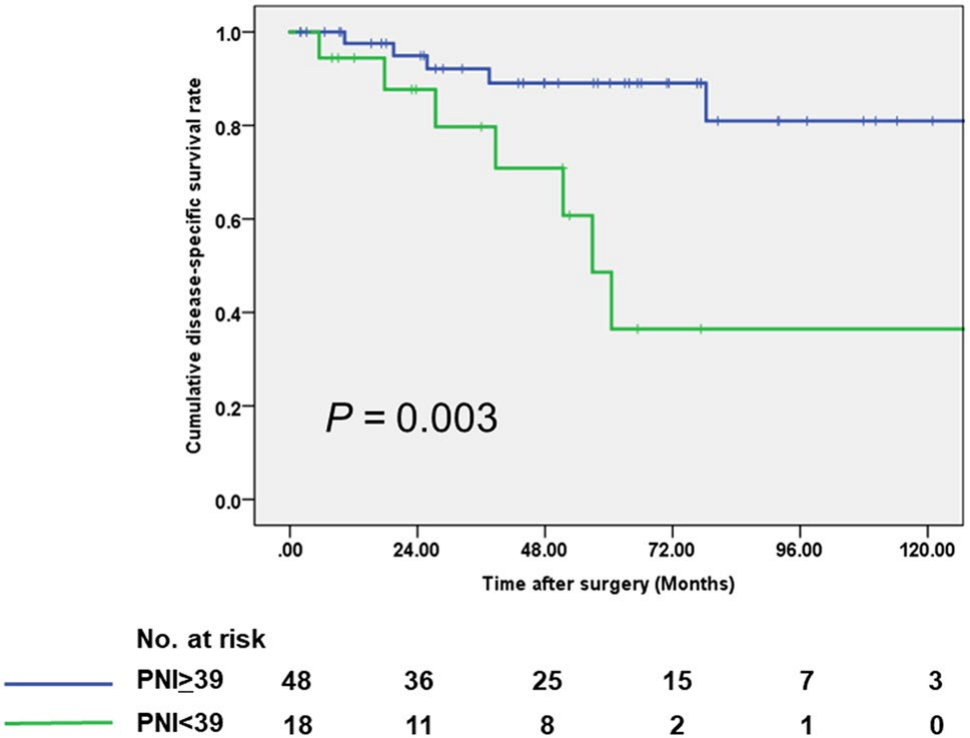
Disease-specific survival classified by the level of PNI in the IPMC patients who underwent pancreatectomy using the Kaplan–Meier method.

Among only preoperative factors, the multivariate analysis revealed that CA19-9 > 22.5 U/mL (hazard ratio [HR] 7.92, 95% CI 1.01–62.4, *P* = 0.049) and PNI < 39 (HR 3.67, 95% CI 1.14–11.9, *P* = 0.030) remained a significant independent predictor of the DSS (Table 4). The cumulative DSS rate of the patients with low PNI was significantly poorer than that of the patients with high PNI (Fig. 1, *P* = 0.003).

**Table 4:** Preoperative prognostic factors for the disease-specific survival in patients with invasive IPMC.

Variables	Univariate		Multivariate	
	Hazard ratio (95% confidence interval)	*P*	Hazard ratio (95% confidence interval)	*P*
Age (> 66 years)	2.50 (0.55–11.4)	0.238		
Sex (males)	0.85 (0.26–2.85)	0.796		
CEA (> 2.5 U/mL)	1.97 (0.58–5.61)	0.275		
CA19-9 (> 22.5 U/mL)	11.0 (1.42–85.1)	0.022	7.92 (1.01–62.4)	0.049
NLR (> 1.74/≤ 1.74)	4.38 (0.95–20.2)	0.058		
PLR (< 70/≥ 70)	2.24 (0.67–7.46)	0.188		
PNI (< 39/≥ 39)	5.08 (1.58–16.4)	0.007	3.67 (1.14–11.9)	0.030
Jaundice, presence	5.69 (1.79–18.1)	0.003		
Enhanced solid component, presence	0.96 (0.61–1.49)	0.841		
Main pancreatic duct, dilatation ≥ 10 mm, presence	0.71 (0.19–2.61)	0.601		

*IPMC* intraductal papillary mucinous carcinoma, *CEA* carcinoembryonic antigen, *CA* carbohydrate antigen, *NLR* neutrophil-to-lymphocyte ratio, *PLR* platelet-to-lymphocyte ratio, *PNI* prognostic nutritional index.

Including pathological and postoperative factors, the multivariate analysis revealed that only lymph node metastasis (LNM) (hazard ratio 11.6, 95% CI 1.03–130.8, *P* = 0.047) remained a significant independent predictor of the DSS (Supplementary Table 1). The cumulative DSS rate of the patients with LNM was significantly poorer than that of the patients without LNM (*P* < 0.001).

### The clinicopathological factors were compared between the patients with and without LNM

The comparison of the clinicopathological factors between the patients with and without LNM was shown in Supplementary Table 2. The CA19-9 level and the rate of jaundice onset were significantly higher in the patients with LNM compared to those without LNM (*P* = 0.001 and *P* = 0.008, respectively). The PNI value was significantly lower in the patients with LNM compared to those without LNM (*P* < 0.001).

### The predictors for the presence of LNM

To determine the cut-off values of continuous variables (CA19-9 and PNI) for predicting the presence of LNM, we used a receiver operating characteristic (ROC) curve and Youden’s index. The ROC curve showed a CA19-9 of 22.5 U/mL with a sensitivity of 81.0%, a specificity of 66.7% and an area under the curve (AUC) of 0.748, and a PNI of 43.5 with a sensitivity of 95.2%, specificity of 50.0% and an AUC of 0.773.

The multivariate analysis showed that a PNI < 43.5 (OR 16.1, 95% CI 1.88–138.5, *P* = 0.011) and CA19-9 level > 22.5 U/mL (OR 6.64, 95% CI 1.73–25.6, *P* = 0.006) were significant predictors of the presence of LNM (Table 5).

**Table 5:** Preoperative predictors for the presence of lymph node metastases.

Variables	Univariate		Multivariate	
	Odds ratio (95% confidence interval)	*P*	Odds ratio (95% confidence interval)	*P*
CA19-9 (> 22.5 U/mL)	8.50 (2.43–29.7)	0.001	6.64 (1.73–25.6)	0.006
PNI (< 43.5)	20.0 (2.47–162.3)	0.005	16.1 (1.88–138.5)	0.011
Jaundice, presence	6.00 (1.69–21.4)	0.006		

*CA* carbohydrate antigen, *PNI* prognostic nutritional index.

### Preoperative scoring system for predicting the presence of LNM

**Figure 2:**
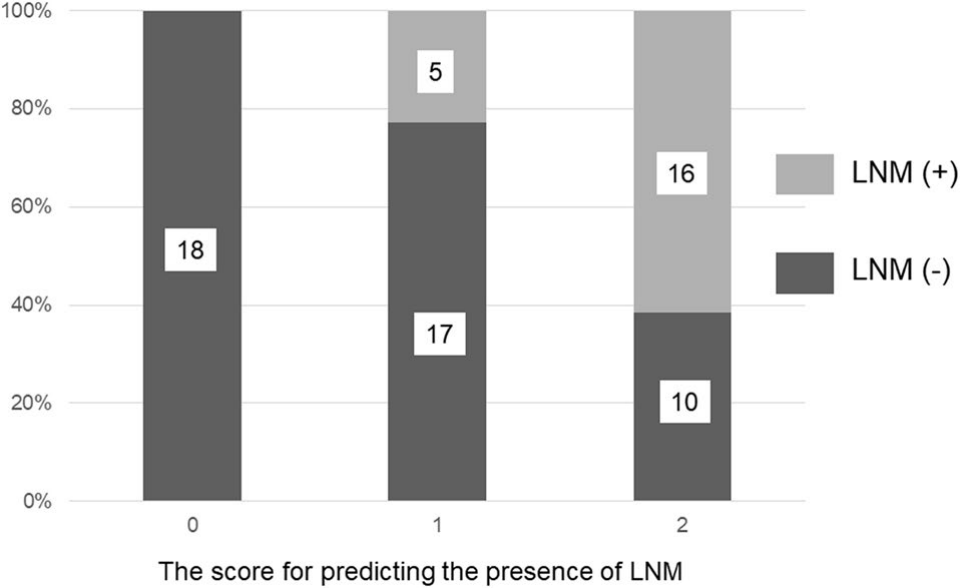
The relationship between the score for predicting the presence of LNM and the rate of actual LNM. The incidence of LNM significantly increased as the score increased (*P* < 0.001).

To establish the preoperative scoring system predicting the presence of LNM, the preoperative CA19-9 level and PNI values, which were easily obtained by preoperative blood tests, were selected. Each risk factor was defined as 1 point and 18, 22 and 26 patients were classified into score of 0, 1 and 2, respectively. The patients with a score of 0 had no LNM. The incidence of LNM significantly increased as the score increased (*P* < 0.001, Fig. 2).

The 5-year DSS rate in the patients with scores of 0, 1 and 2 were 100%, 90% and 57.6%, respectively. The DSS of the patients with a score of 2 was significantly poorer than that of the patients with scores of 0 and 1 (*P* = 0.007 and *P* = 0.026, respectively).

## Discussion

The present study showed that PNI value as well as CA19-9 were significant predictors of invasive IPMC among the preoperative factors. The present study suggested the prognostic and diagnostic roles of PNI value for the IPMN patients.

Several reports have described prognostic factors of invasive IPMC^19–21^, including the rate of an invasive component^19^, perineural invasion^19^, distant metastasis^19^, lymph node ratio^21^, CA19-9^21^ and family history of pancreatic cancer^21^ as independent prognostic factors. Among the prognostic factors of invasive IPMC, there are few that can be identified before surgery. Preoperative PNI value is therefore an important role of deciding the treatment strategy for IPMN patients because it has the correlation with diagnostic ability for predicting malignant IPMN and prognosis.

The definitive diagnosis of LNM, except in cases where bulky LNM findings are present on the preoperative images, is extremely difficult because the findings of lymphadenopathy in IPMN do not reflect the presence of LNM despite predicting malignancy of IPMN^22^. If the presence of LNM can be accurately determined preoperatively, surgeons can select the neoadjuvant chemotherapy. However, there were no descriptions in any guidelines as to the treatment strategy for the patients with advanced-stage IPMC^23,24^.

The present study showed that LNM was not observed and the DSS rate was 100% in the patients with a score of 0 (27% of invasive IPMC patients). In contrast, LNM was observed in more than half of patients with a score of 2, and the 5-year DSS rate in this group was around 50%. The DSS rate was markedly poorer in those patients than in patients with scores of 0 and 1, but was slightly better than in cases of PDAC. These results suggest that minimally invasive surgery, like omitting lymph node dissection, might be considered for patients with low CA19-9 and high PNI values. In contrast, similar to PDAC, pancreatectomy with systematic lymph node dissection is desirable for patients with high CA19-9 and low PNI values. Scoring system including PNI value may therefore play an important role in making decisions regarding operative procedures.

Our scoring system for predicting the presence of LNM involves two factors: CA19-9 and the PNI. CA19-9 is one of the strong prognostic factors for PDAC^12^ and is reported as a malignant predictive factor^25^ and prognostic predictor^21^ in IPMN. However, the PNI as a predictor of LNM in invasive IPMC is a novel finding, as this has not been previously reported in the field of IPMN.

The PNI was first reported by Onodera in Japan, as a nutritional status combining the albumin level and lymphocyte count^26^. The cut-off value of PNI as 45 was originally proposed as a determinant of the surgical indication, not as a prognostic factor^26^. On the other hand, the cut-off value was defined to be 39 and 43.5 using an ROC curve in the present study. As a result, we successfully selected a patient group with a good prognosis, accounting for 27% of invasive IPMC patients, by combining the PNI with the CA19-9 level.

Although there has been no comprehensive study on the relationship between the PNI and LNM in the field of pancreatic tumors, a meta-analysis was performed for patients with gastric cancer to explore the prognostic role of the PNI and the relationship between the PNI and clinicopathological characteristics^27^. In that analysis, a low PNI was found to have a significant relationship with the presence of LNM. Furthermore, the circulating tumor cell number, which is a feasible way of monitoring the tumor cell spread, had a negative correlation with a decreased PNI in patients with gastric cancer^28^. Patients with invasive IPMN often develop jaundice and cholangitis, and as a result, the PNI value as well as serum albumin level might be decreased in these patients. Moreover, LNM may lower serum albumin level, resulting in a lower PNI, as observed with gastric cancer^28^. Further studies are needed to clarify why the PNI was a predictor of the presence of LNM in patients with invasive IPMC. The PNI value, like gastric cancer, may reflect the spread of tumor cells in invasive IPMC.

A limitation of the present study is its retrospective in nature and was performed at a single center. Further prospective multi-institutional studies are therefore needed to objectively validate the results of the present study. Moreover, although the OR differed between CA19-9 and PNI, the scoring system developed without considering the weights of the two predictor for the presence of LNM because the range of 95% CI was wide and thus the strength of the OR was doubtful. And the cut-off value of CA19-9 for predicting the malignant IPMN and the presence of LNM was 22.5 U/mL, which was within the normal range. Finally, it has been still difficult to diagnose invasive IPMC correctly. However, we believe that the results of the present study have a certain value for the patients with high-risk stigmata according to the Fukuoka criteria^3^.

In conclusion, we suggested the prognostic and diagnostic roles of PNI value in the IPMN patients. Our novel scoring system combining CA19-9 and PNI values clearly clarified the rate of LNM and the prognosis, suggesting that this system is useful for making decisions of regarding operative procedures for the IPMN patients.

## Methods

### Patients and methods

The patients who underwent pancreatectomy for IPMN at the Division of Hepato-Biliary-Pancreatic Surgery, Shizuoka Cancer Center, between October 2002 and December 2017, were included in the study. This study confirmed to the ethical guidelines of the World Medical Association Declaration of Helsinki-Ethical Principles for Medical Research Involving Human Subjects. Written informed consent for surgery and use of patients’ clinical data was obtained from all patients who were included in the present study. We obtained approval from the Institutional Review Board of Shizuoka Cancer Center (approval number: J2019-41-2019-1-3). The present study excluded the IPMN patients with concurrent conventional PDAC. The pathologist (SK) performed the diagnosis from the resected specimens according to the 2010 WHO criteria^29^. Although the 2010 WHO criteria defined high-grade dysplasia (carcinoma in situ) as a benign neoplasm, such patients were classified as a malignant neoplasm in the present study because the diagnostic abilities of systemic inflammation responses and nutritional status for predicting the malignant potential tumor in which surgery should be considered was evaluated.

Diagnostic procedures and indications for surgery were previously mentioned^6^. In brief, the diagnosis of IPMN was reached using a combination of diagnostic modalities such as enhanced computed tomography (CT) and abdominal ultrasonography. Endoscopic ultrasound was not routinely performed for patients with IPMN. Select cases were examined using a combination of multiple investigational modalities, such as positron emission tomography, endoscopic retrograde cholangiopancreatography and biopsy. Regarding the indications for surgery, until the publication of Sendai criteria, our indications for surgery highly depended on the decision of the individual staff surgeon due to the absence of guidelines. After the publication of Sendai and Fukuoka criteria, the indications for surgery adhered to these criteria in principle and were ultimately decided by the cancer board.

The NLR was calculated by dividing the absolute neutrophil count (/mm^3^) by the absolute lymphocyte count (/mm^3^), the PLR was calculated dividing the absolute platelet count (/mm^3^) by the absolute lymphocyte count (/mm^3^) and the PNI was calculated as the albumin (g/L) + 0.005 × the absolute lymphocyte count (/mm^3^).

Variables including the sex, age, tumor markers, and indexes of a systemic inflammation response and nutritional status, including NLR, PLR and PNI, were collected. At imaging, the following data were recorded from the findings of the CT examination, as all patients underwent CT in the present study: cyst size, diameter of main pancreatic duct (MPD), and the presence of an enhanced solid component. The cyst size was the same as MPD diameter in the patients with main duct-type IPMN. The tumor stage was assessed based on the seventh edition of the Union Internationale Contra le Cancer classification^30^.

All patients were evaluated every three months until postoperative 2 years, and physical examinations, blood tests and follow-up contrast-enhanced CT or ultrasonography were performed. After postoperative 2 years, these examinations were performed every three to six months according to the malignant potential. When a recurrence was identified, chemotherapy was routinely introduced. The follow-up period for the analysis of DSS ended at the time of death due to IPMN. The remaining patients were treated as censored case at the last follow-up visit. The study period ended in March 2018.

### Statistical analyses

Continuous data are presented as the median and range and were compared using the Mann–Whitney *U* test. The categorical variables were employed using χ^2^ tests or Fisher’s exact probability tests, as appropriate. To identify the significant predictors of malignant IPMN, the multivariate analysis was performed using the logistic regression method with a backward stepwise selection model.

The Kaplan–Meier method was applied for analyzing the cumulative DSS and the survival value was compared by the log-rank test. A Cox proportional hazards model was used for the univariate analysis, and variables with *P* < 0.05 in the univariate analysis were entered into the multivariate analysis. To identify the possible predictors of malignant IPMN and significant predictors of the DSS and the presence of LNM, the multivariate regression analysis was performed using a backward stepwise selection model. Statistical analyses were performed using the SPSS 24.0 software package (SPSS, Inc., Chicago, IL, USA), and *P* values of < 0.05 in two-tailed tests were considered statistically significant.

### Ethical approval

This study confirmed to the ethical guidelines of the Declaration of Helsinki (2013 revision) and was retrospective in nature, and we obtained approval from the Institutional Review Board of Shizuoka Cancer Center for the exception of patient consent (approval number: J2019-41-2019-1-3).

## Supplementary Information

Supplementary Table 1.

Supplementary Table 2.
