# Supplementary material for: The prognostic roles of the prognostic nutritional index in patients with intraductal papillary mucinous neoplasm
Source: Sci Rep. 2021 Jan 12;11:568. doi: 10.1038/s41598-020-79583-6 (PMC7803756; doi:10.1038/s41598-020-79583-6)
Supplement: Supplementary file 1 — Supplementary Table 1. [file 41598_2020_79583_MOESM1_ESM.docx]

**The prognostic roles of the prognostic nutritional index in patients with intraductal papillary mucinous neoplasm**

Yukiyasu Okamura, MD, PhD, FACS, Teiichi Sugiura, MD, PhD, Takaaki Ito, MD, PhD, Yusuke Yamamoto, MD, PhD, Ryo Ashida, MD, PhD, Katsuhisa Ohgi, MD, Hiroto Narimatsu, MD, PhD, Keiko Sasaki, MD, Katsuhiko Uesaka, MD, PhD

| **Supplementary Table 1** Prognostic factors for the disease-specific survival in patients with invasive IPMC | | | | |
| --- | --- | --- | --- | --- |
| Variables | Univariate | | Multivariate | |
|  | Hazard ratio  (95% Confidence interval) | *P* | Hazard ratio  (95% Confidence interval) | *P* |
| Age (>66/<66 years) | 2.50 (0.55-11.4) | 0.238 |  |  |
| Sex (males/females) | 0.85 (0.26-2.85) | 0.796 |  |  |
| CEA (>2.5/<2.5 U/mL) | 1.97 (0.58-5.61) | 0.275 |  |  |
| CA19-9 (>22.5/<22.5 U/mL) | 11.0 (1.42-85.1) | 0.022 |  |  |
| NLR (>1.74/<1.74) | 4.38 (0.95-20.2) | 0.058 |  |  |
| PLR (<70/>70) | 2.24 (0.67-7.46) | 0.188 |  |  |
| PNI (<39/>39) | 5.08 (1.58-16.4) | 0.007 |  |  |
| Adjuvant chemotherapy (not performed/performed) | 0.98 (0.26-3.62) | 0.973 |  |  |
| Venous invasion (presence/absence) | 4.14 (1.29-13.2) | 0.017 |  |  |
| Lymphatic invasion (presence/absence) | 30.0 (3.81-236) | 0.001 |  |  |
| Neural invasion (presence/absence) | 26.6 (3.38-209) | 0.002 |  |  |
| Serosal invasion (presence/absence) | 1.16 (0.15-9.10) | 0.888 |  |  |
| Retroperitoneal invasion (presence/absence) | 2.87 (0.86-9.58) | 0.087 |  |  |
| T factor (1+2/3) | 2.17 (0.28-16.9) | 0.458 |  |  |
| Lymph node metastasis (presence/absence) | 39.7 (5.08-309.8) | <0.001 | 11.6 (1.03-130.8) | 0.047 |
| IPMC: intraductal papillary mucinous carcinoma; CEA: carcinoembryonic antigen; CA: carbohydrate antigen; NLR: neutrophil-to-lymphocyte ratio; PLR: platelet-to-lymphocyte ratio; PNI: prognostic nutritional index. | | | | |
