# Supplementary material for: The prognostic roles of the prognostic nutritional index in patients with intraductal papillary mucinous neoplasm
Source: Sci Rep. 2021 Jan 12;11:568. doi: 10.1038/s41598-020-79583-6 (PMC7803756; doi:10.1038/s41598-020-79583-6)
Supplement: Supplementary file 2 — Supplementary Table 2. [file 41598_2020_79583_MOESM2_ESM.docx]

**The prognostic roles of the prognostic nutritional index in patients with intraductal papillary mucinous neoplasm**

Yukiyasu Okamura, MD, PhD, FACS, Teiichi Sugiura, MD, PhD, Takaaki Ito, MD, PhD, Yusuke Yamamoto, MD, PhD, Ryo Ashida, MD, PhD, Katsuhisa Ohgi, MD, Hiroto Narimatsu, MD, PhD, Keiko Sasaki, MD, Katsuhiko Uesaka, MD, PhD

| **Supplementary Table 2** Preoperative characteristics of invasive IPMC patients with and without LNM | | | |
| --- | --- | --- | --- |
|  | With LNM | Without LNM | *P* |
|  | n=21 | n=45 |  |
| Age (years) ^#^ | 68 (36-82) | 70 (52-86) | 0.549 |
| Sex (males/females) | 16/5 | 32/13 | 0.772 |
| CEA (U/mL)^#^ | 3.1 (0.5-17.0) | 2.6 (0.5-15.7) | 0.778 |
| CA19-9 (U/mL)^#^ | 50 (2-706) | 11 (2-2,661) | 0.001 |
| Cyst diameter (mm)^#^ | 36 (20-73) | 40 (11-140) | 0.469 |
| Main pancreatic duct diameter (mm)^#^ | 6.5 (3.0-50.0) | 8.0 (3.0-25.0) | 0.583 |
| NLR | 1.87 (1.10-6.12) | 2.02 (0.98-8.85) | 0.863 |
| PLR | 77.1 (35.9-242.6) | 74.0 (31.8-256.8) | 0.695 |
| PNI | 38.2 (23.1-44.2) | 43.7 (22.1-50.1) | <0.001 |
| Operative indication according to the Fukuoka criteria, presence | 21 (100) | 44 (97.8) | 1.000 |
| High-risk stigmata according to the Fukuoka criteria, presence | 21 (100) | 40 (88.9) | 0.313 |
| Jaundice, presence | 9 (42.9) | 5 (11.1) | 0.008 |
| Enhanced solid component, presence | 20 (95.2) | 36 (80.0) | 0.150 |
| Main pancreatic duct, dilatation ≥10 mm, presence | 6 (28.6) | 18 (19.0) | 0.422 |
| IPMN type according to the Fukuoka criteria |  |  | 0.134 |
| Main duct | 3 | 17 |  |
| Branch duct | 11 | 15 |  |
| Mixed | 7 | 13 |  |
| The findings of suspected LNM on preoperative images |  |  |  |
| Ultrasonography | 2 (9.5) | 1 (2.2) | 0.337 |
| Computed tomography | 4 (19.1) | 3 (6.7) | 0.189 |
| Values in parentheses are percentages unless otherwise indicated.  Values are shown as the ^#^median (range)  IPMC: intraductal papillary mucinous carcinoma; LNM: lymph node metastases; CEA: carcinoembryonic antigen; CA: carbohydrate antigen; NLR: neutrophil-to-lymphocyte ratio; PLR: platelet-to-lymphocyte ratio; PNI: prognostic nutritional index; IPMN: intraductal papillary mucinous neoplasm | | | |
